# A Smart Colorful Supercapacitor with One Dimensional Photonic Crystals

**DOI:** 10.1038/srep18419

**Published:** 2015-12-22

**Authors:** Cihui Liu, Xing Liu, Hongyun Xuan, Jiaoyu Ren, Liqin Ge

**Affiliations:** 1 State Key Laboratory of Bioelectronics, School of Biological Science and Medical Engineering, Southeast University, Nanjing 210096, China

## Abstract

To meet the pressing demands for portable and flexible equipment in contemporary society, developing flexible, lightweight, and sustainable supercapacitor systems with large power densities, long cycle life, and ease of strongly required. However, estimating the state-of-charge of existing supercapacitors is difficult, and thus their service life is limited. In this study, we fabricate a flexible color indicative supercapacitor device with mesoporous polyaniline (mPANI)/Poly(N-Isopropyl acrylamide-Graphene Oxide-Acrylic Acid) (P(NiPPAm-GO-AA)) one dimensional photonic crystals (1DPCs) as the electrode material through a low-cost, eco-friendly, and scalable fabrication process. We found that the state-of-charge could be monitored by the structural color oscillation due to the change in the photonic band gap position of the 1DPCs. The flexible 1DPCs supercapacitor is thin at 3 mm and exhibits good specific capacitance of 22.6 F g^−1^ with retention of 91.1% after 3,000 cycles. This study shows the application of the 1DPCs supercapacitor as a visual ultrathin power source. The technology may find many applications in future wearable electronics.

Flexible energy storage devices such as electrochemical capacitors are the best candidates for energy storage devices that can be operated in bio-environments because it is easier to find biocompatible components and to exclude the electrochemical reactions for supercapacitors than for batteries or fuel cells^1^. However, the present supercapacitors could not meet the demand of high energy densities and convenient use kind^2^,^3^,^4^,^5^,^6^. It is difficult for us to estimate the state-of-charge of existing supercapacitors which leads to blind and excessive charge/discharge thus limiting their service lives^7^. So, it is significant to introduce a visual supercapacitor which can indicate the state-of-charge and extend the supercapacitor life.

Nature offers a remarkably simple alternative idea that can offer a stacking structure with visible structural color^8^. Recently, it was found that one dimensional photonic crystals (1DPCs) can demonstrate a well stacking structure with vivid structural color, and the colors could be the monitor of supercapacitors’ charge/discharge process^9^. Hence, it is useful to construct a similar stacking structure to imitate the natural 1DPCs to study them directly in man-made systems.

Polyaniline (PANI) is a well-studied conducting polymer for supercapacitors due to its high specific capacitance from multiple doping/dedoping states although it often suffers from inferior long-term stability and rate capability due to the swelling and shrinking during the rapid charge/discharge process^10^. More interestingly, the mesoporous structure can effectively solve the problem. Compared with pristine PANI, mesoporous PANI (mPANI) electrode exhibits dramatically improved specific capacitance which can enhance the rate performance and the cyclic stability^11^.

Graphene oxide (GO), with active oxygen functionalities on their planes facilitate surface, has aroused considerable interest as a new electron transport material in recent materials science and condensed-matter studies^12^. The irreversible capacitance which can be considered as an increase of inert components in graphene-based supercapacitors is mainly caused by the two-dimensional stack of graphene and the reduced conductivity of electrons or protons arising therefrom^13^,^14^. Aggregation and stacking problems are commonly observed in most of the free-standing graphene papers reported to date^15^,^16^,^17^. As a result, the unique properties of individual graphene sheets such as high accessible surface area are compromised or not achievable in the macroscopic graphene paper. In order to improve the capacitance, we fabricate P(NiPAAm-GO-AA) hydrogel. It take advantage of GO’s superior electrochemical properties and electron transport ability.

Furthermore, in previous studies the degradation of specific capacitance was mainly due to severe swelling and shrinking of PANI during the charge/discharge process, which may cause mechanical degradation and breaking of the chains^18^,^19^,^20^. To date, most of the covalent connections in the PANI/GO composite materials which were used to make supercapacitors were individually based on the functional groups of GO or PANI^6^,^21^. Therefore, efforts to enhance the synergistic effect between PANI and GO to improve the overall performance of PANI/GO supercapacitors are still needed^22^. Note that PANI was widely studied to form composites with carbon nanomaterials for supercapacitors in both two-electrode and three-electrode systems, but the specific capacitors when put mesoporous PANI with hydrogel P(NiPPAm-GO-AA) are not seen yet, so the smart 1DPC supercapacitor was novel which the charging and discharging process can be observed by visible structural color change.

In this paper, we have developed a flexible mPANI/P(NiPPAm-GO-AA) 1DPC supercapacitor. This supercapacitor can make visible color change during the charge/discharge process to indicate the state-of-charge. These characteristics may extend the life of the supercapacitor. The flexible 1DPC supercapacitor is thin at 3 mm, and exhibits good specific capacitance of 22.6 F g^−1^ with retention of 91.1% after 3,000 cycles.

## Results

### Fabrication of a flexible mPANI/P(NiPPAm-GO-AA) 1DPC supercapacitor

**Figure 1:**
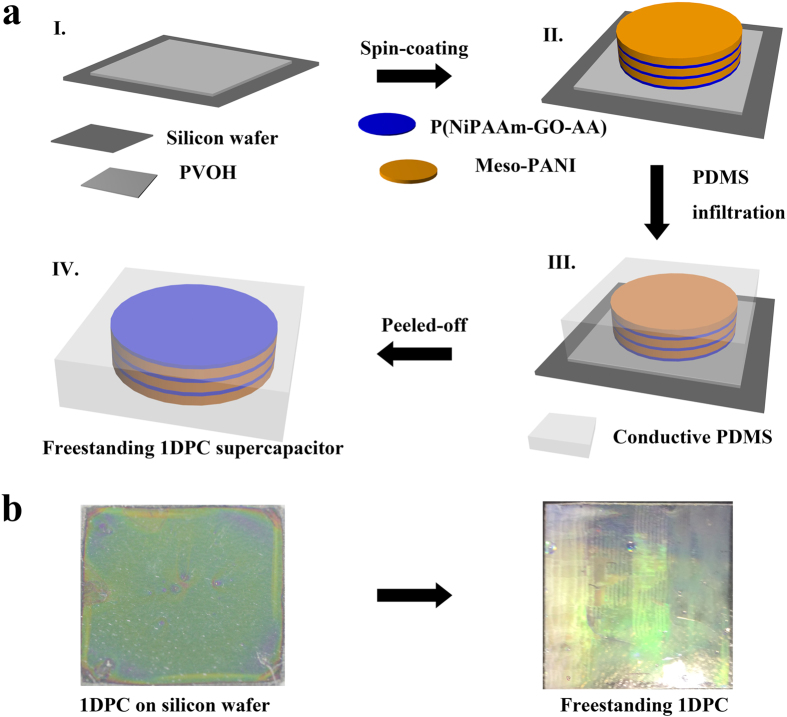
(**a**) Fabrication of flexible 1DPCs supercapacitor (I-IV) Schematic illustration showing the freestanding flexible 1DPCs supercapacitor fabricating process. (I) A Polyvinyl alcohol (PVOH) film is placed on a silicon wafer. (II) A P(NiPAAm-GO-AA)/mPANI 1DPC is spin-coated onto PAOH silicon wafer. (III) A PDMS overcoat is added onto 1DPC to create a planar capping layer. (IV) The freestanding flexible 1DPCs supercapacitor is peeled-off from silicon wafer after PVOH layer was dissolved. (**b**) The digital photographs of the 1DPC on silicon wafer and freestanding 1DPC with vivid structural color.

The fabrication of the flexible supercapacitor is schematically shown in Fig. 1a. A thin layer of water soluble sacrificial layer Polyvinyl alcohol (PVOH) film was coated onto a silicon wafer. The PVOH layer was then baked at 110 °C for 1 min to evaporate residual water. The 1DPC structural mPANI/ P(NiPAAm-GO-AA) were spin-coated onto the PVOH layer^23^. Then, the conducting PDMS film was placed top down onto the 1DPCs. To make conducting PDMS, we modified PDMS with conducting carbon blacks filler. The interfacial contact area between the 1DPC film and PDMS also influences the efficiency of the transfer process. After 20 min bake treatment, the PDMS film with mPANI/P(NiPAAm-GO-AA) Bragg stack structure was peeled off from the silicon wafer substrate when PVOH layer was dissolved. The flexible supercapacitor is finally fabricated by assembling 1DPC with conducting flexible PDMS, Fig. 1b.

### Characterizations of a flexible mPANI/P(NiPPAm-GO-AA) 1DPC supercapacitor

The fabrication of mesoporous polyaniline (mPANI/P(NiPAAm-GO-AA)) 1DPCs was conducted by alternating mPANI and P(NiPAAm-GO-AA) hydrogel through spin-coating. PANI is one of the most promising conducting polymers for supercapacitor application due to its high conductivity, unique redox properties and easy synthesis approaches. A facile approach has been developed to fabricate mPANI through *in situ* polymerization using mesoporous silica composite as template. The mesoporous structure can effectively solve the problem that PANI often suffers from inferior long-term stability and rate capability due to swelling and shrinking during the rapid charge/discharge process. Compared with pristine PANI, mPANI electrode exhibits dramatically improved specific capacitance, enhanced rate performance and cyclic stability in 1 mol L^−1^ H_2_SO_4_^24^.

P(NiPAAm-GO-AA) is formed through the solution radical copolymerization of NiPAAm, GO and AA with BIS as the initiator in deionized water. The unique hydrogel structure of the GO satisfies several critical requirements for an ideal electrode. The GO in hydrogel network is interconnected and interlocked to prevent it from restacking and to maintain a highly porous monolithic graphene framework. The effective refractive indexes of the P(NiPAAm-GO-AA) and mPANI layers were determined by spectroscopic ellipsometry to be 1.63 and 1.33 respectively. The structural color naturally offers an indication of the lattice structure of the photonic crystal. Color changes can be visually tracked by the naked eye.

**Figure 2:**
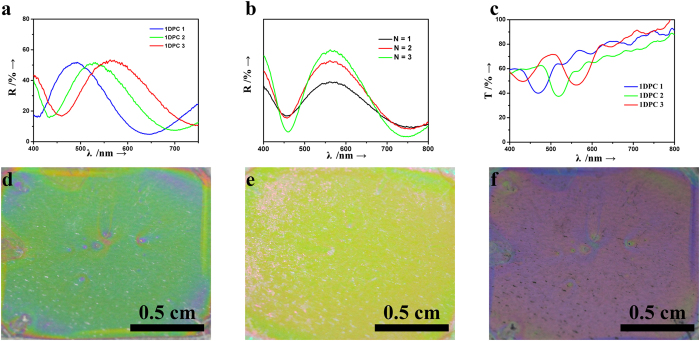
Optical properties of flexible 1DPCs supercapacitor. (**a**) The reflection spectra of 1DPCs with different mPANI thickness. (**b**) The reflection spectra of a 1DPC with different bilayer numbers. (**c**) Then transmission spectra of 3 different flexible 1DPCs. (**d**–**f**) The digital images of 3 different 1DPCs film with different structural color.

The tuning of optical properties of 1DPCs can be achieved by increasing the number of layers, as shown in Fig. 2. An intense and wide Bragg peak (with peak position at 575 nm) is observed; the outcome is a direct consequence of the large refractive index contrast between the mPANI layer and the polymer layer. The photonic band width becomes narrower and the intensity of the peak grows with increasing numbers of layers. The result is in good agreement with the previous theory of the thickness dependence of the optical response of photonic crystal slabs (Fig. 2a).

A change in layer thickness also influences the optical properties of 1DPCs. By increasing the layer thickness, the position of the band gap will make a red shift because of Bragg-Snell’s law. According to the significant refractive index difference between two components, the obvious photonic stopband and structural color can be easily obtained in several bilayers. As shown in the reflective spectra (Fig. 2a), the Bragg peak positions are shown to be at 480, 530 and 580 nm, respectively, by fixing the thickness of P(NiPAAm-GO-AA) layer at a proper thickness while changing the thickness of mPANI layer increasingly. The finding agrees with the experiment results. The Bragg peak positions stay unchanged when increasing the numbers of bilayers while keeping the thickness constant (Fig. 2b). The Bragg peak can be manipulated in the full visible range from green (Fig. 2d) to yellow (Fig. 2e) and red (Fig. 2f) by choosing proper periods. Based on reflection spectra characteristics of the mPANI response with electron transfer between emeraldine salt (ES) and emeraldine base (EB) forms, we designed a flexible 1DPC supercapacitor with various stopbands that overlap the whole range of visible reflectance spectra.

The corresponding transmittance spectra are plotted in Fig. 2c, along with the spectra of the multilayers in the PDMS template. The resultant thin Bragg stack sheets were transparent, with optical transmittances at over 90%. As designed, the ordered mPANI/ P(NiPAAm-GO-AA) 1DPCs were stacked to enable high electrical conductivity necessary for the high performance of the resultant supercapacitor (Fig. 2d–f). The film was very uniform over a large area. In our experiments, color uniformity can be achieved over an area of 15 mm  × 15 mm at least. The 1DPCs could maintain its color to be invariable for a long time. This ability indicates that the prepared materials and microstructures have perfect stability.

**Figure 3:**
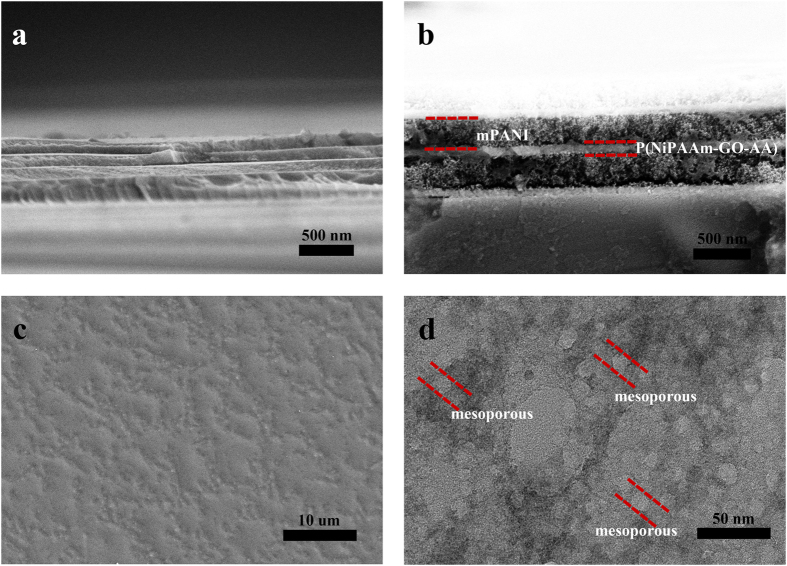
SEM and TEM images. (**a**) SEM image of a cross section view of a 1DPC film; (**b**) SEM image of enlarged view of a flexible 1DPC film; (**c**) SEM image of top view of P(NiPAAm-GO-AA) layer; (**d**) TEM image of synthesized mesoporous PANI.

A series of scanning electron microscopy (SEM) and transmission electron microscopy (TEM) images is shown in Fig. 3 to further understand the preparation process of the 1DPC mPANI/ P(NiPAAm-GO-AA) architecture. Figure 3a shows a cross-section view of the 1DPC film. A typical flexible capacitor indicates that the mPANI and P(NiPAAm-GO-AA) hydrogel is in a well-ordered stack arrangement in the PDMS substrate. The PDMS flexible capacitors were peeled off from the silicon wafer substrate that had been synthesized through spin coating. The 1.0 μm-thick Bragg stacks structure could be stably attached onto the flexible PDMS film. It could be peeled off from the substrate traced by the SEM even after many charge/discharge cycles (Fig. 3b). The fracture edge of the film reveals compact layer-by-layer stacking throughout the entire cross-section. The stacking of laminated GO was inhibited by introducing NiPAAm and AA to form a P(NiPAAm-GO-AA) hydrogel microstructure, which shows a clear stratification microstructure of P(NiPAAm-GO-AA) (Fig. 3c). As expected, PANI was well stacked in the 1DPCs to produce high-quality composite films. The mPANI/P(NiPAAm-GO-AA) 1DPC retained the aligned structure even at a mPANI weight percentage of 35% to 60%.

The 1DPC has bright structural colors, which indicate the prominent optical properties of the supercapacitor. For the pure GO template, the top surface of the P(NiPAAm-GO-AA) free-standing film was uniformly covered by a large quantity of small and compact mesoporous PANI. As shown in the cross-section SEM image of the mPANI, no mPANI was observed in the inner layers of the P(NiPAAm-GO-AA) sheets. This phenomenon clearly suggests that for the pure P(NiPAAm-GO-AA) film, the mPANI was stacked in a compact manner on the surface of the GO hydrogel film because of the stacked lamellar structure. Remarkably, a high-resolution TEM image shows that the resultant mPANI sheets had an extremely flat shape and numerous mesoporous structures with size of approximately 10 nm (Fig. 3d). The BET test is in supplementary information Figure S2. The cross-section SEM images clearly demonstrate that the mPANI and P(NiPAAm-GO-AA) have been successfully stacked into a whole flexible PDMS template from the top surface, down to the interior of the architecture.

### Electrochemical evaluation of the mPANI/P(NiPAAm-GO-AA) supercapacitor.

To investigate the electrochemical performance of mPANI/P(NiPAAm-GO-AA) in a real device, a symmetric supercapacitor device was constructed using mPANI/P(NiPAAm-GO-AA) as the working electrode and Platinum (Pt) as the counter electrode. The good capacitance of mPANI/ P(NiPAAm-GO-AA) 1DPCs films may be attributed to the internal microstructure of the deposited mPANI. This phenomenon can be explained by the decreasing conductivities along with the increasing PANI weight percentage^25^. In consideration of the specific capacitance, the PANI weight percentage of 60% is investigated in the following discussion.

According to a previous study, graphene/PANI supercapacitors cannot provide an effective network for electron transportation during electrochemical reaction if they are stacked loosely^26^. To solve this problem, we made a well-stacked mPANI/P(NiPAAm-GO-AA) 1DPC structure, in which the hydrogel P(NiPPAm-GO-MAA) made the stack dense.

**Figure 4:**
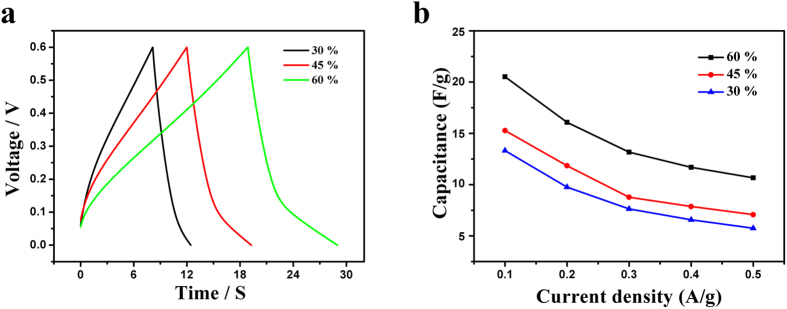
(**a**) Galvanostatic charge-discharge profiles of supercapacitors with different mPANI weight percentages; (**b**) Dependence of specific capacitance on mPANI weight percentage and current density.

The galvanostatic charge/discharge curves of the obtained mPANI/P(NiPAAm-GO-AA) in different densities were measured to further study their possible application as supercapacitor electrode materials. The typical galvanostatic charge/discharge curves of the mPANI/P(NiPAAm-GO-AA) electrode in 1 M H_2_SO_4_ are presented in Fig. 4. Charge-discharge curves maintain the same shape at a potential range of 0 to 0.7 V in these densities. Therefore, the nanocomposite can experience a wide range of current. Moreover, the discharge/charge efficiency can be obtained according to the ratio of the discharge time to the charge time.

The resultant supercapacitor exhibited a high electrochemical performance. Figure 4a compares the galvanostatic charge/discharge profiles of the supercapacitors based on different mPANI weight percentages at 0.1 A g^−1^. The maximal specific capacitance was 60%. To further explore the influence of the PANI weight percentage on the specific capacitance, galvanostatic charge/discharge processes were also conducted in increasing current densities from 0.1 to 0.5 A g^−1^.

As shown in Fig. 4b, the specific capacitances were gradually enhanced from 13.8 F g^−1^ to 22.6 F g^−1^, with the increasing mPANI weight percentage due to the improved capacitance from the PANI. With the increasing current densities from 0.1 A g^−1^ to 0.5 A g^−1^, the capacitances were maintained 70.1%, 71.3% and 74.5% at the PANI weight percentages of 30%, 45% and 60%, respectively.

The different mPANI percentage 1DPC film electrodes can be also divided into different thicknesses of the mPANI layer varies from 50 nm to 150 nm. The specific capacitance was increased with the increasing mPANI thickness because of the decreasing resistance along the aligned direction. The influence on the percentage of mPANI in smart supercapacitor is summarized in Fig. 4. A positive electrode was examined to consider the symmetric structure in the supercapacitor. Conducting polymers were shown to change structures under different oxidation states^27^.

**Figure 5:**
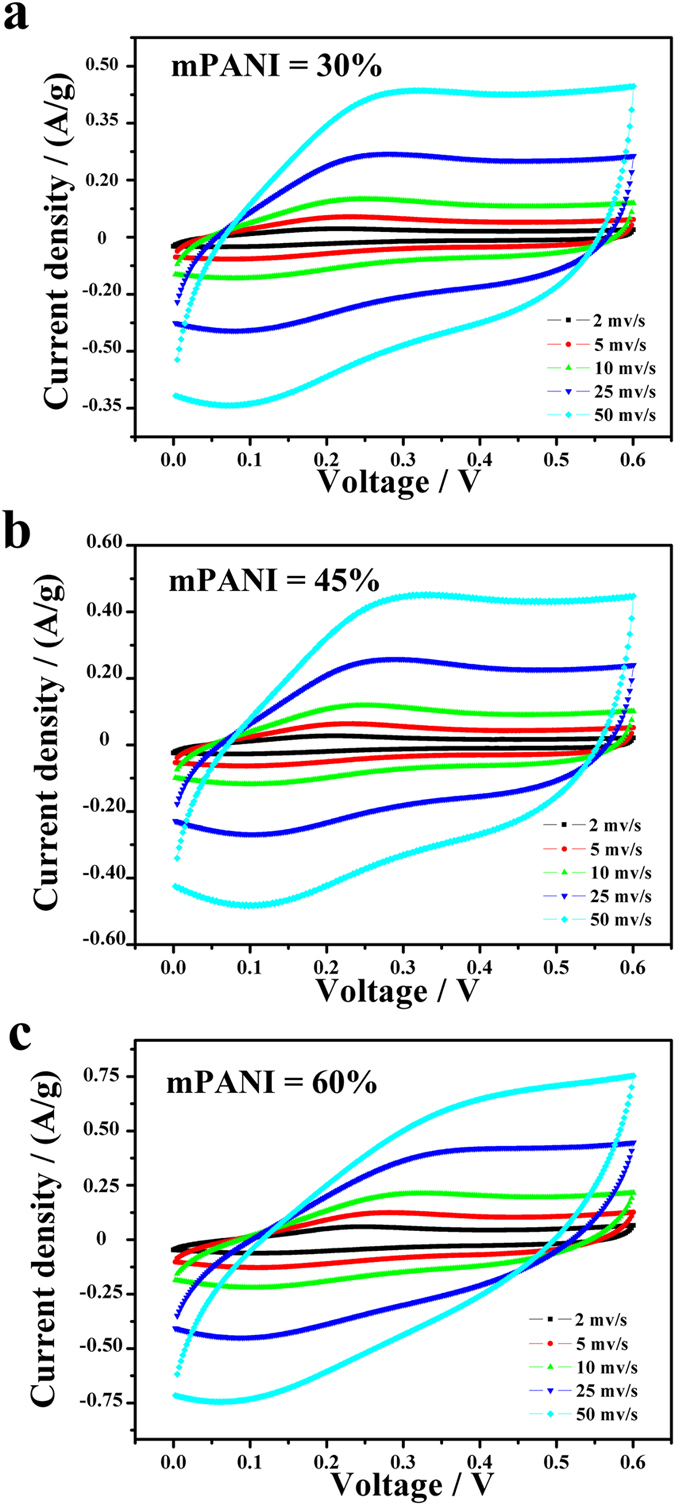
Cyclic voltammograms of different mPANI weight percentage with increasing scan rates. (**a**) mPANI weight percentage of 30%; (**b**) mPANI weight percentage of 45%; (**c**) mPANI weight percentage of 60%.

Figure 5 shows the representative CVs for three different tandem PANI weight percentages (30%, 45%, and 60%) at different scan rates of 2, 5, 20, 25, and 50 mV s^−1^ in 1 M H_2_SO_4_. The rate-dependent cyclic voltammograms of the electrode over a wide range of scan rates are shown in Fig. 4 and they exhibit a typical redox behavior of PANI. The shape of the CV was maintained even up to the high scan rate of 50 mV s^−1^, This result indicates that the mPANI/P(NiPAAm-GO-AA) 1DPCs film has a rapid charge/discharge response within the potential window of 0.6 V in the device^22^. Normally, pure PANI shows two couples of redox peaks: one is due to the transition of PANI from the semiconducting-state to the conductive form, and the other is due to the transition from emeraldine to pernigraniline. The GO electrode also exhibits a pair of peaks that originated from the oxygenous groups on the surface. However, when GO and mPANI are combined into a flexible 1DPC mPANI/P(NiPAAm-GO-AA), their CV curves become different from each component electrode and exhibit the characteristics of both double-layered capacitance and pseudocapacitance. More interestingly, the CV shapes of different PANI percentage change with different mass ratios. As the binder of each working electrode is inactive and occupies the same amount, the change in CV characteristic is due to the nature of the 1DPC materials. The synergetic effect resulting from the interactions of PANI and GO may affect the shape and potential position of the CV curves.

The existence of peak on each CV plot for the samples indicates the existence of the faradic processes. The charge storage in the faradic process is achieved by electron transfer that leads to chemical changes in the electroactive materials. The redox peaks can be ascribed to the changing in mPANI structures and the oxygenated groups attached to the surface of the graphene oxide nanostructures. Besides, the surface chemical functional groups significantly influence the interfacial capacitance by introducing pseudocapacitance as depicted in former works. Thus, graphene oxide sheets with different amounts in the composites may play an important role in determining the CV shapes.

Synthesized materials also exhibit excellent electrochemical behavior in a wide range of scan rates (2 mV s^−1^−50 mV s^−1^) in H_2_SO_4_ electrolyte solution. For example, for mPANI percentage of 30% (Fig. 5a), 45% (Fig. 5b) and 60% (Fig. 5c), the current density of both samples increases with the scan rate and the curve shape is steady, thus indicating the good electrochemical stability of the electrode material. This stability is due to the introduction of the GO hydrogel with a high mechanical property into the 1DPC and the synergetic effect between the two components. The CV shape of 30% mPANI is different from that of 60% mPANI probably because of the different feeding ratios and the different Faradic charge transfer reactions arising from the interaction and synergy among individual components.

The synthesis of PANI nanocomposites and their application in energy storage have aroused great research interests recently. The 1DPC stack structure with mPANI we prepared effectively increased the specific surface area. The increase is one of the reasons for the high specific capacitance. The microstructures of 1DPC films were significantly influenced by the weight percentage of mPANI, which plays a key role during the fabrication of the capacitor. The further increase in mPANI weight percentage led to the increase in 1DPC capacity. However, as expected, the optical transmittance decreased with the increased thickness of the mPANI sheet. Thus, the 60% weight percentage of the mPANI sheet with thickness of 150 nm was mainly studied in this work.

**Figure 6:**
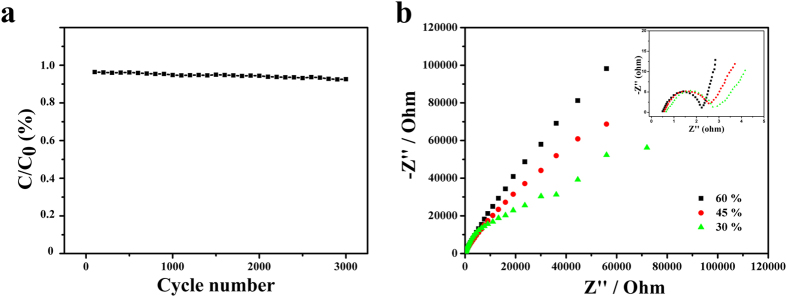
(**a**) Dependence of specific capacitance on cycle number at a current density of 0.5 A/g; (**b**) Nyquist plots of 3 different mPANI weight percentage flexible electrodes. Inset is the enlarged plots of the high-frequency region.

To further understand the electrochemical stability of the mPANI/P(NiPAAm-GO-AA) electrode, consecutive charge–discharge cycles were measured at a current density (Fig. 6a). The mPANI/P(NiPAAm-GO-AA) electrode was shown to maintain 91.1% of its initial capacitance after 3000 cycle tests. The finding suggests that the mPANI/P(NiPAAm-GO-AA) electrode exhibits excellent long-term cycle ability and a high degree of reversibility in consecutive charge/discharge cycles. Therefore, the mPANI/P(NiPAAm-GO-AA) 1DPC is shown to have good cyclic stability as a supercapacitor electrode material^28^.

The mPANI/P(NiPAAm-GO-AA) electrode exhibits high discharge/charge stability, which indicates excellent discharge/charge rate control capability and electrochemical reversibility. PANI commonly suffers from poor cyclic stability as an electrode material for supercapacitors because of the cyclic mechanical stress caused by material swelling and shrinking during the charge/discharge cycle. The enhanced electrochemical performance can be firstly attributed to the introduction of GO hydrogel into the 1DPC material and mesoporous structure. This Bragg stack synthesis procedure enables the resultant supercapacitors to be less susceptible to fatigue caused by cyclic mechanical stress.

Electrochemical impedance spectroscopy (EIS) is an important tool for the study of the characteristics of conductivity, mechanistic analysis of interfacial processes and structure and charge transport at the material electrolyte interface. Figure 6b shows the Nyquist diagrams for PANI, P(NiPAAm-GO-AA) and the resultant mPANI/ P(NiPAAm-GO-AA) 1DPC. The semicircle in the high-frequency region and the straight line in the low-frequency region correspond to the electron-transfer-limited processes and diffusional-limited electron-transfer processes, respectively. The diameter of the semicircle is one of the limiting factors for the power density of supercapacitors, as it corresponds to the charge transfer resistance at the electrode-electrolyte interface. Compared with that of pure PANI, the charge transfer resistance of the mPANI/P(NiPAAm-GO-AA) electrode is reduced remarkably because of the formation of the hierarchical nanocomposite due to the use of P(NiPAAm-GO-AA) as a support. In the low frequency, the slope of the 45^°^ portion of the Nyquist plots is called the Warburg resistance (R_w_) and is a result of the frequency dependence of ion diffusion in the electrolyte on the electrode interface. The R_w_ value for the 60% mPANI is larger than that of the 30% and 45% mPANI electrode, which demonstrates a better accessibility of the electrolyte ions to the 30% and 45% mPANI electrode. These EIS results support our CV analyses of the enhanced performance of the flexible electrode.

**Figure 7:**
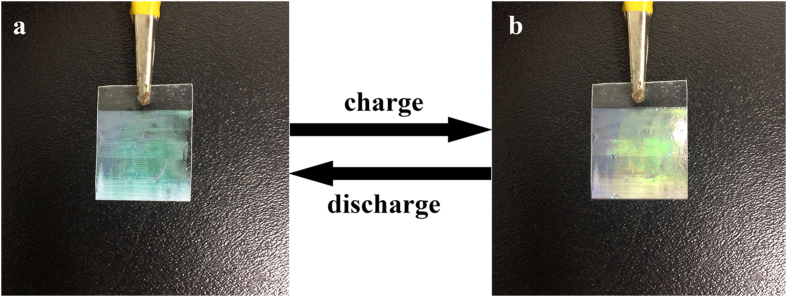
Structural color change of flexible 1DPCs supercapacitor during charging-discharging process. The 1DPCs film make a red shift from green (**a**) to yellow (**b**) after a charging process.

Interestingly, the supercapacitor exhibits different colors during the charge diacharge process caused by the shift of the 1DPC stopbands. The structural color changes because of the variation of the refractive index of the mPANI in different charge/discharge processes (Fig. 7). The 1DPCs film make a red shift from green (Fig. 7a) to yellow (Fig. 7b) after a charging process, as the mPANI changes to the fully conducting form and the refractive index increases (Supporting Information Fig. S3 and Movie [SI]). By contrast, the 1DPC film turns back to green after the discharge process as the refractive index decreases. The effective refractive index of the mPANI is obtained by spectroscopic ellipsometry. Upon a full charge with a voltage of 0.6 V, the electrode turns yellow. When the supercapacitor is discharged to 0.6 V, the electrode changs to green.

This phenomenon indicates that the supercapacitor under the charge/discharge process can be monitored by the naked eyes. Although PANI has been widely explored in various sensing materials including electrochromatic nanocomposites, to the best of our knowledge, this work represents a better combination of chromatic devices and supercapacitors using PANI.

## Discussion

This attractive supercapacitor is attributed to the Bragg stack with tunable structural color and a good combination of a mesoporous conducting polymer and GO hydrogel. These flexible mPANI/P(NiPAAm-GO-AA) 1DPCs supercapacitors can be an ideal candidate for state-of-charge monitoring application because of their distinct color change under charge/discharge process.

The good supercapacitive performance of mPANI/P(NiPAAm-GO-AA) 1DPCs may be largely attributed to their unique structure. First, 1DPCs that consist of mesoporous PANI have high porosity, and thus the electrolyte can easily penetrate through the Bragg stack for efficient redox reactions during the charge storage process. Second, they offer an ordered lamellar structure when GO is combined with the P(NiPAAm-GO-AA) hydrogel. Third, the unique Bragg stack structure can significantly enlarge the active surface area and improve the structural integrity as well. As a result, the stare-of-charge of a flexible 1DPC supercapacitor can be monitored by the structural color change, and the specific capacitance and cycling stability are enhanced.

In summary, a novel smart and colorful supercapacitor with high performance is developed in this study. This supercapacitor can make visible color changes during the charge/discharge process to indicate the state-of-charge. These characteristics may extend the life of the supercapacitor. The flexible 1DPC supercapacitor is thin at 3 mm and exhibits good specific capacitance of 22.6 F g^−1^ with retention of 91.1% after 3,000 cycles. This work can serve as a starting point in designing smart supercapacitors with 1DPCs, which cannot be done with their conventional counterparts. We demonstrate the application of the 1DPC supercapacitor as an ultrathin power source. This technology may find extensive applications in future wearable electronics.

## Method

### Synthesis of mPANI and P(NiPPAm-GO-AA).

All the chemicals were directly used after purchase without further purification. In a typical synthesis, the mesoporous PANI was uniformly synthesized on the surface of mesoporous SiO_2_ (mSiO_2_) via *in situ* polymerization. The mSiO_2_ was achieved from Nanjing Dongjian Biological Technology Co., LTD. 0.47 g of mSiO_2_ was dispersed in 40 mL of deionized water and stirred for 2 h. Then 20 mL of aniline monomers (solvent: 1 mol L^−1^ HCl) with the concentration of 0.25 mol L^−1^ was added into the above mixed solution. After stirred for another 2 h, 20 mL of ammonium persulfate solution (the same concentration as that of aniline monomers) was slowly added into the above solution and the system temperature was controlled below 4 ^o^C in an ice-water bath. As the reaction proceeded, the color of the solution gradually turned emerald. After the reaction continued for 4 h, the mSiO_2_/PANI was obtained after filtration and washing. Finally, silica was removed by the treatment with hydrofluoric acid under magnetic stirring for 24 h and the obtained materials was mPANI. P(NiPPAm-GO-AA) polymer hydrogel was prepared by free-radical polymerization. NIPAM (120.0 mg), AA monomer (3.0 mg), GO (3.0 mg), BIS (12.0 mg) and H_2_O (2.0 mg) were mixed in a flask. Meanwhile, nitrogen gas was also purged into the solution for 30 min to remove dissolved oxygen. The solution was stirred at 350 rpm for 4 h at 70 ^o^C. As a result, P(NiPPAm-GO-AA) polymer hydrogel was formed as white suspension in aqueous phase.

### Fabrication of flexible mPANI/P(NiPPAm-GO-AA) 1DPCs supercapacitor.

A thin layer of water soluble sacrificial layer PVOH film was coated onto a silicon wafer. The PVOH layer was then baked at 110 °C for 1 min to evaporate residual water. The 1DPC structural mPANI/ P(NiPAAm-GO-AA) were spin-coated onto the PVOH layer. Then, the conducting PDMS film was placed top down onto the 1DPCs. To make conducting PDMS, we modified PDMS with conducting carbon blacks filler. The interfacial contact area between the 1DPC film and PDMS also influences the efficiency of the transfer process. After 20 min bake treatment, the PDMS film with mPANI/P(NiPAAm-GO-AA) Bragg stack structure was peeled off from the silicon wafer substrate when PVOH layer was dissolved. The flexible supercapacitor is finally fabricated by assembling 1DPC with conducting flexible PDMS.

### Structural characterization and analysis

Field emission scanning electron microscope (FESEM, Zeiss Ultra Plus) was used to see the structure of the flexible 1DPCs supercapacitor. Transmission electron microscope (TEM, JEM2100EX) was used to obtain the image of mSiO_2_ and mPANI. Reflective spectra were recorded using a fiber optic UV-vis spectrometer (Ocean Optic HR2000CG).

### Electrochemical measurements

All the electrochemical experiments were carried out using Autolab PGSTAT302N (Metrohm Autolab, Netherlands) at room temperature. The electrical impedance spectroscopy (EIS) measurements were performed at open circuit potential with a sinusoidal signal over a frequency range from 100 kHz to 10 mHz at an amplitude of 10 mV. The cycle life tests were conducted by galvanostatic charge/discharge measurements. Galvanostatic charge–discharge measurements of half cells, symmetric supercapacitors and hybrid devices were carried out galvanostatically at various current densities with voltage windows specific to the materials using a battery test system Chi660D (Chenhua Inc., Shanghai, China).

The gravimetric capacitances (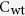) of flexible 1DPCs electrodes derived from galvanostatic discharge curves were calculated based on the following equation 1:


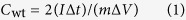


where 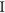 is the constant discharge current, 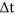 is the time for a full discharge, 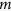 is the net mass of one electrode and 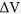 represents voltage drop on discharging (excluding the *V*_*drop*_)^29^,^30^

The spectral position of the reflection maximum, 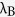 of a multilayer made of two types of alternating films of thicknesses 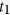 and 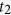 and refractive indices 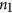 and 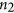, respectively, depends on the incident angle with respect to the surface normal, 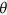, as expressed in the equation 2:


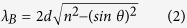


Where *d* is the unit cell size and n is the average refractive index of the multilayer, which is in turn given by equation 3:


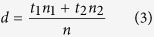


For more 1DPCs theoretical equation in Supplementary Information.

## Additional Information

**How to cite this article**: Liu, C. *et al.* A Smart Colorful Supercapacitor with One Dimensional Photonic Crystals. *Sci. Rep.* **5**, 18419; doi: 10.1038/srep18419 (2015).

## Supplementary Material

supporting information

Supplementary Movie
