# Supplementary material for: A Smart Colorful Supercapacitor with One Dimensional Photonic Crystals
Source: Sci Rep. 2015 Dec 22;5:18419. doi: 10.1038/srep18419 (PMC4686878; doi:10.1038/srep18419)
Supplement: supporting information [file srep18419-s1.doc]

**Electronic Supplementary Information**

**A Smart Colorful Supercapacitor with One Dimensional Photonic Crystals**

Cihui Liu, ^1^ Xing Liu, ^1^ Hongyun Xuan, ^1^ Jiaoyu Ren, ^1^ and Liqin Ge ^1^*

^1^ State Key Laboratory of Bioelectronics, School of Biological Science and Medical Engineering, Southeast University, Nanjing 210096, P.R. China

*To whom correspondence should be addressed. Email: lqge@seu.edu.cn

**Supplementary Movies**

**Movie** shows the state of charge/discharge process of the 1DPCs. The structure color change can be obtained through the process. It is not easy to catch the color change when 1DPCs is immersed into the electrolyte solution, so we took it out and photograph the 1DPCs which makes it easier to see.

**Supplementary Materials**

**Bragg-Snell equation:**

The PBGs of the 1DPCs could be estimated from the Bragg-Snell equation:

*m*λ_Bragg_=2*D(n*^2^_eff_– *sin*^2^*θ)*^1/2^ (1)

*n*^2^_eff_= *n*_1_^2^*f*_1_ + *n*_2_^2^*f*_2_ (2)

Where *m* is the diffraction order, D is the period, λ_Bragg_ is the position of Bragg peak, *θ* is the incident angle, *n*_eff_ is the effective refractive index, *and n_1_*, *f_1_, n*_2_ and *f_2_* are refractive indexes and volume fractions of the two different layers respectively.

**Refractive index and stop bands**


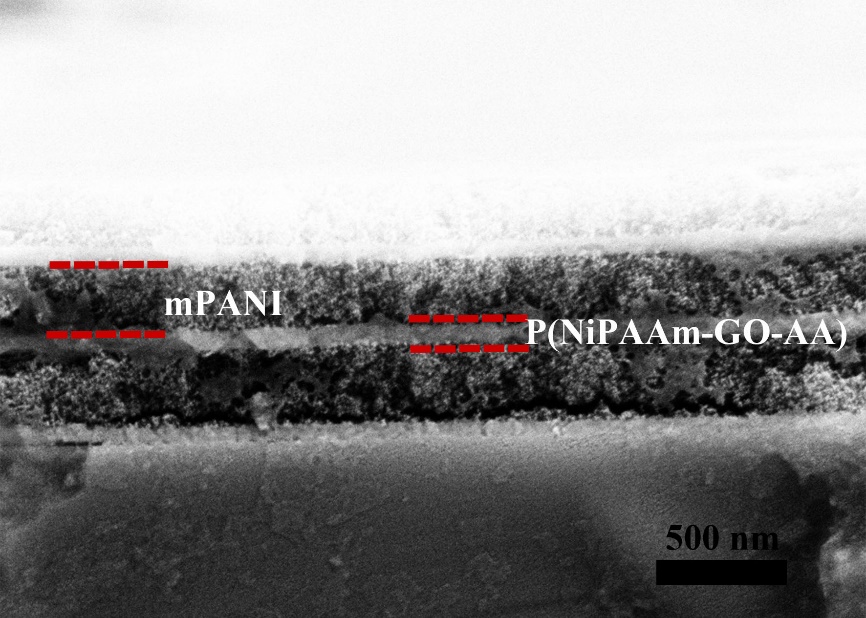


**Figure S1**. SEM image of a 1DPCs

According to the formula (1) and (2) above, we can calculate the theoretical value of the photonic stop bands. The effective refractive index of the mPANI layer was determined by spectroscopic ellipsometry to be 1.33. The effective refractive index of the P(NiPAAm-GO-AA) layer was determined by spectroscopic ellipsometry to be 1.63. The average thicknesses of P(NiPAAm-GO-AA) layer and mPANI layer are 100 nm and 350 nm respectively which were observed by SEM, **Figure S1**. The stopband of this 1DPC is at about 530 nm.

**BET characterization of mPANI**

We also did the BET characterization. The BET surface area is 805.1 m^2^/g, and the porosity is 0.70 cm^3^/g, as **Figure S2**.


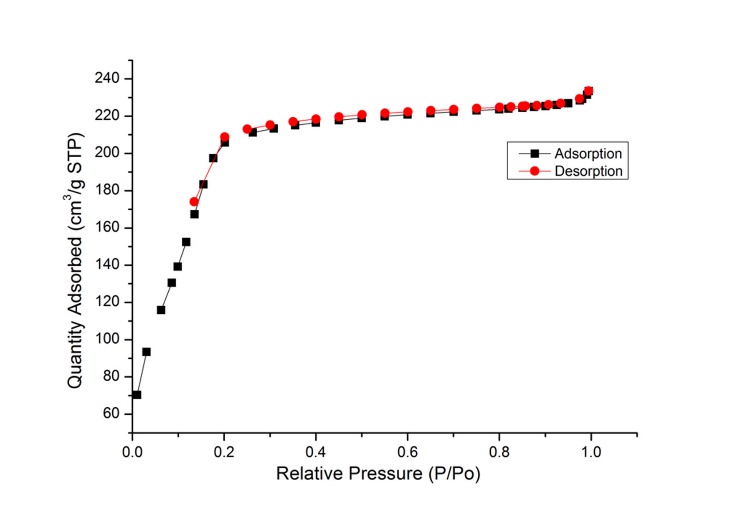


**Figure S2**. The BET characterization of mPANI.

**In-situ color change**

The in-situ photographs of the 1DPCs under charge-discharge process is in **Figure S3**. As Fig. S3 shows, the film color made a red shift from blue to green. We also made a movie to record the process, but it is also not easy to catch the color change when put it in the electrolyte solution. As image shows, from 1 to 2 is the charge process, the film color made a red shift, 2 to 3 is the discharge process, the film color made a blue shift and 3 to 4 is the charge process, the film color made a red shift.


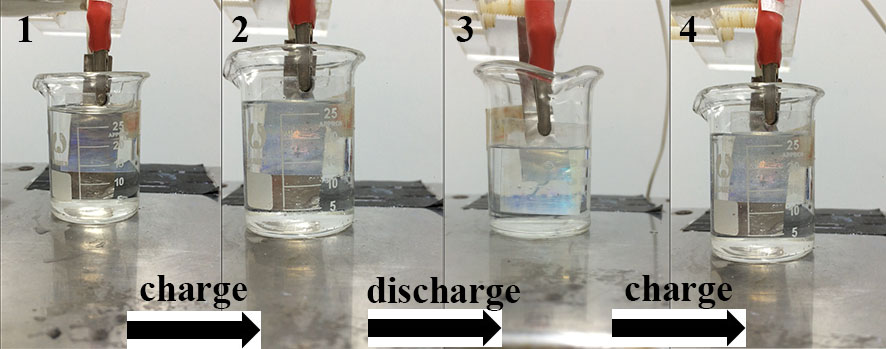


**Figure S3**. The in-situ photographs of the 1DPCs under charge-discharge process. As image shows, from 1 to 2 is the charge process, 2 to 3 is the discharge process, and 3 to 4 is the charge process.
